# Nurse-Driven mHealth Implementation Using the Technology Inpatient Program for Smokers (TIPS): Mixed Methods Study

**DOI:** 10.2196/14331

**Published:** 2019-10-04

**Authors:** Amanda C Blok, Rajani S Sadasivam, Timothy P Hogan, Angela Patterson, Nicole Day, Thomas K Houston

**Affiliations:** 1 Veterans Affairs Center for Clinical Management Research Veterans Affairs Ann Arbor Healthcare System United States Department of Veterans Affairs Ann Arbor, MI United States; 2 Systems, Populations and Leadership Department School of Nursing University of Michigan Ann Arbor, MI United States; 3 Division of Health Informatics and Implementation Science Department of Population and Quantitative Health Sciences University of Massachusetts Medical School Worcester, MA United States; 4 Veterans Affairs Center for Healthcare Organization and Implementation Research Veterans Affairs Bedford Medical Center United States Department of Veterans Affairs Bedford, MA United States; 5 University of Massachusetts Memorial Health Center Worcester, MA United States; 6 Learning Health Systems Department of Medicine Wake Forest University Winston-Salem, NC United States

**Keywords:** implementation strategy, telemedicine, mHealth, tobacco use cessation, care transition, patient transfer, smoking cessation, mobile health, smoking, tobacco

## Abstract

**Background:**

Smoking is the leading cause of preventable death and disease, yet implementation of smoking cessation in inpatient settings is inconsistent. The Technology Inpatient Program for Smokers (TIPS) is an implementation program designed to reach smokers with a mobile health (mHealth) intervention using stakeholder-supported strategies.

**Objective:**

The purpose of this study was to determine the impact of the TIPS implementation strategies on smoker-level engagement of the mHealth intervention during care transition.

**Methods:**

We examined varying intensities (passive motivational posters only and posters + active nurse-led facilitation) of TIPS strategies on four hospital units located in two sites. Unit-level and smoker-level adoption was monitored during active implementation (30 weeks) and sustainability follow-up (30 weeks). Process measures reflecting the reach, effectiveness, adoption, implementation, maintenance (RE-AIM) framework, stakeholder reported adaptations of strategies, and formative evaluation data were collected and analyzed.

**Results:**

For our smoker-level reach, 103 smokers signed up for the mHealth intervention in-hospital, with minimal decline during sustainability follow-up. While posters + nurse facilitation did not lead to higher reach than posters alone during active implementation (27 vs 30 signed up), it did lead to higher engagement of smokers (85.2% vs 73.3% completion of the full 2-week intervention). TIPS strategy adoption and fidelity varied by unit, including adoption of motivational posters (range: weeks 1 and 5), fidelity of posters (0.4% to 16.2% of posters missing per unit weekly) and internal facilitation of nurse training sessions (average of 2 vs 7.5 by site). Variable maintenance costs of the program totaled US $6.63 (US $683.28/103) per smoker reached. Reported family-member facilitation of mHealth sign-up was an observation of unintended behavior.

**Conclusions:**

TIPS is a feasible and low-cost implementation program that successfully engages smokers in an mHealth intervention and sustains engagement after discharge. Further testing of nurse facilitation and expanding reach to patient family and friends as an implementation strategy is needed.

## Introduction

Tobacco use is the primary preventable cause of death and disease in the United States [1]. Treating smoking-related illness costs $170 billion in direct medical costs and over $156 billion in lost productivity [1,2]. Furthermore, tobacco use is a major risk factor for many chronic illnesses that commonly result in hospitalization, such as cancer, heart and lung diseases, chronic obstructive pulmonary disease, and diabetes [1]. Patients who resume smoking upon discharge are more likely to be rehospitalized [3]. Hospitalization is a unique period of forced abstinence, and this is an opportunity to engage smokers and motivate them to be smoke free as they transition home after discharge [1]. However, continual engagement with tobacco cessation support can be a challenge when transitioning away from the clinical setting.

Mobile technology allows for on-hand support and is often willingly ported by users. More than 9 out of 10 smokers in the United States own a mobile phone, and the majority of patients in the hospital setting can access text-enabled phones [4,5]. The application of mobile health (mHealth) technology has been recognized as an evidence-based approach to tobacco cessation since 2011 [6], yet programs to connect smokers with this technology have not been implemented in hospitals.

While tobacco cessation interventions have been effective [7], they are often challenging for hospitals to implement and sustain [8]. While intensive interventions involving nurse-administered toolkits have increased abstinence [9], they are difficult to integrate into hospital staff workflow, leaving a gap in dissemination across hospital settings. Further, these nurse-administered interventions infrequently engage smokers after discharge. Hospital managers across the United States report a perceived lack of action addressing tobacco use in the hospital setting [10]. Hospital staff members face numerous barriers to addressing patient tobacco use, including time constraints, inadequate support, and limited training in tobacco cessation counseling [11,12]. Hospitals need implementation programs designed to address these barriers [8].

We developed an implementation program called the Technology Inpatient Program for Smokers (TIPS) to support the use of an mHealth intervention designed to continue engaging smokers during transition into the outpatient setting [13]. TIPS is a low-cost program that lets managers and staff employ intuitive, theory-driven strategies to reach people using a technology-supported behavioral intervention. The TIPS program consists of multiple strategies: (1) motivating mHealth intervention use through a promotional poster campaign and (2) activating nursing staff to facilitate patient sign-up. The purpose of this study was to determine the adoption and fidelity of TIPS strategies by units and measure smokers’ engagement in the mHealth intervention.

## Methods

### Study Design

TIPS was evaluated at two sites using a phased implementation study design, with purposeful increases in intensity of implementation strategies. Each phase followed a standard operating procedure for all nursing units. The aims of our evaluation were to compare the following outcomes across phases:

- Implementation fidelity, adoption, cost, and nurse stakeholder experiences
- Impact of the implementation program on smoker engagement with the mHealth intervention

TIPS had three phases: two active implementation phases (15 weeks each) and one phase of sustainability monitoring (30 weeks). Clinical units received a different intensity of implementation strategies in the two active implementation phases (passive motivational posters only and posters + active nurse-led facilitation) to determine potential benefits of additional implementation strategies. We hypothesized that the active nurse-led inpatient strategy would result in higher postdischarge smoker engagement with the mHealth intervention compared with posters only. These two active 15-week phases were followed by a sustainability phase. To achieve our aims, we used mixed methods to determine the feasibility of the study design, implementation program, and patient engagement. These data were collected in preparation for a larger implementation trial. The study was approved by the University of Massachusetts Medical School institutional review board.

### Implementation Framework

The implementation program design was guided by the Practical and Robust Implementation Science Model (PRISM) [14]. We selected PRISM out of many emerging implementation science theoretical frameworks because of its particular focus on challenges of evidence-based practice integration in the clinical setting and its integration of the diffusion of innovations theory to guide the uptake of technology-assisted interventions. PRISM guided our formative work and our effectiveness assessment using the reach, effectiveness, adoption, implementation, and maintenance (RE-AIM) framework [15].

### Setting and Sample

Four hospital units were recruited from two Northeast sites in the same network. We asked hospital leadership representatives to identify units containing high proportions of patients with chronic cardiac and pulmonary conditions. Since tobacco use heavily affects these prevalent conditions, the program could greatly benefit this population [16,17]. Nurse unit manager buy-in was obtained through meetings in which a nurse facilitator presented the program, assessed stakeholders’ organizational perspectives as outlined by the PRISM model [14], and constructed a plan for communication between the facilitator and stakeholders during implementation.

### Effectiveness Data-Supported Intervention

The TIPS implementation program supports the use of the effectiveness data-supported mHealth technology intervention: the texting system. Over the course of two weeks, the system delivered motivational messages written by smokers for smokers, encouraging participants to abstain from smoking [18]. These messages were created using current clinical guidelines [19] and social cognitive theory [20] and evaluated in the outpatient setting through a Web-assisted tobacco intervention [13,21-23]. This outpatient evaluation, a multisite randomized controlled trial with 900 smokers, found that motivational messages increased 6-month smoking cessation outcomes (odds ratio 1.7; 95% CI 1.0-2.8) compared with controls [13]. For our study, messages not appropriate for use in the inpatient setting (ie, “take a walk outside”) were removed from the message protocol.

### Implementation Program

**Figure 1:**
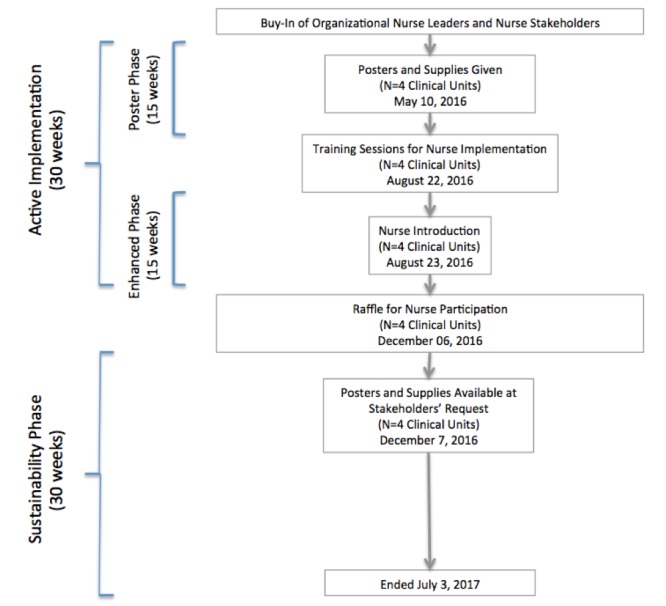
Study flow diagram.

TIPS included 30 weeks of active implementation and 30 weeks of sustainability monitoring for a total 60-week duration (Figure 1). Stakeholders including leaders, managers, and staff participated in a formative assessment leading to the final design of the TIPS program [24]. Weekly contact with nurse managers allowed feedback collection. Shared decision-making for adaptations to program strategies were elicited and typically implemented the following week.

#### Poster Phase

Active implementation included two phases of varying intensities. An initial 15-week phase, the poster phase, reflected the lowest program. All clinical units received promotional posters for use at the discretion of nurse unit managers. Poster content was created using health belief model concepts [24,25]. Posters were designed to motivate smokers to sign up for the texting system using their mobile phones. Communications and material safety hospital boards approved the posters. Managers received an informational letter containing guideline-based instructions for secure posting and optimal placement and additional materials to hang posters as requested throughout the study.

At the end of the poster phase (15 weeks), two training sessions for nurses on each unit were performed by external facilitators (nurse scientists with clinical nurse specialist certification) to explain the TIPS program to staff nurses. The nurse training sessions were adapted with permission from a previously evaluated nurse education session for tobacco cessation [26]. Through nurse stakeholder feedback, education sessions were shortened to less than 10 minutes and presented during existing staff training times. Thereafter, nurse unit managers continued to train their staff.

#### Enhanced Phase

A second 15-week phase, the enhanced phase, used additional strategies for nurse-driven facilitation of the texting system including a protocol for introducing mobile messages and cue cards with the protocol strategically placed on nurse-specific computer carts. The protocol was shortened to three steps for ease of implementation: (1) ask on admission if the patient is a smoker, (2) point to the poster and give the patient an invitation to sign up, and (3) document the patient’s decision in the electronic health record (EHR).

#### Sustainability Phase

In the 30-week sustainability phase, stakeholders were encouraged to contact the external facilitators to request supplies or communicate needs that arose. The promotional posters remained on patient room walls during the entire study.

### Data Collection and Measures

#### Aim 1. Measures of Implementation Program Evaluation at the Unit and Nurse Level

Characteristics of units were collected from nurse unit managers; these included patient diagnoses and number of beds on the unit. We surveyed nurse unit managers’ perceptions of readiness using the Organizational Readiness for Change Assessment (ORCA) survey [27], measuring three core elements: quality of evidence, environment or context for implementation, and facilitation of the implementation process.

Implementation fidelity, an agents’ fidelity to the various elements of an intervention’s protocol, includes the consistency of delivery of program components [15]. Process evaluation measures were identified from a taxonomy developed by Proctor and colleagues [28] and from a nurse-driven tobacco cessation intervention in the hospital setting by Duffy and colleagues [26]. Poster display fidelity was assessed weekly during active implementation and once during and after the sustainability phase. Poster fidelity was defined as the rate of missing posters by unit at each time point. Key dates when nurse managers facilitated the adopted implementation strategies were recorded for each unit; this reflected other milestone-driven implementation evaluation models [29]. Measuring implementation processes also involves identifying various changes made to strategies [30]. All adaptations to the program based on weekly stakeholder feedback were recorded. Fixed costs and variable costs were summed from administrative documents using a micro-costing system and reported by phase [31]. Interviews with stakeholders identified associated costs beyond these.

Barriers to patient adoption of the mHealth program were collected before and after active implementation by survey of the nurse staff. The question posed to nurses was, “Do you think any of the below reasons could be a barrier to introducing mobile messages to patients?” Responses were a checklist of potential barriers from a previously developed questionnaire [32] and those identified during the formative assessment [24].

Formative evaluation data on the strategies and intervention from purposive samples of nurses were collected by qualitative interview. Interview guides were developed using questions from previous evaluations [32]. We gathered acceptability data, asking the extent to which they agreed with the statement, “I would recommend other health care professionals to introduce these text messages as well.”

#### Aim 2. Measures of Impact of the Implementation Program on Smoker Level Outcomes

Reach refers to “the absolute number, proportion, and representativeness of individuals who are willing to participate in a given initiative” [15]. This was reported as the absolute number of participants who signed up by week over the three phases as recorded in the mobile message system database. The number of smokers admitted to the units was measured during active implementation using EHR database reports designed for tobacco treatment specialist use; these listed current everyday and someday smokers by unit. To assist in examining reach by unit, the mobile message database captured date and content of received messages from smokers, such as self-reported location in the hospital.

Engagement has been used as a proximal measure for behavior change in previously tested digital behavior change interventions [33]. We measured daily engagement using the mobile message system database, with early disengagement of participants indicated by a response text message of “Stop.” In addition to our quantitative outcome, we conducted follow-up interviews with smokers. In parallel to the nurse interviews, guides were developed from previous evaluations [32]. Acceptability was ascertained by asking smokers about their level of agreement with the statement, “I would recommend these text messages for others” [34].

### Statistical Analyses

#### Implementation Program Evaluation at the Unit and Nurse Level

ORCA scores were calculated using a validated procedure, with mean scores reported for each survey category [27]. Poster fidelity was calculated as the rate of missing posters on patient walls by unit (the number of posters missing on units divided by the number of beds on the unit) for each time point. The average poster fidelity was calculated per unit for each phase during active implementation and at one time point during and one time point after the sustainability phase.

#### Nurse Experience With the Program

Perceived and actual barriers to patient participation in the intervention were reported as the percentage of nurses identifying each potential or observed barrier. Participants in TIPS were invited to give feedback on the program at the end of the enhanced phase. Interviews were audio-recorded and transcribed. Interviews were first analyzed using a rapid identification of themes using audio recordings procedure to ascertain feedback needing immediate attention or action [35]. After this, transcripts were analyzed using open-ended coding: researchers reviewed coding together and agreed on the final data display in table format.

#### Impact of the Implementation Program on Smoker Level Outcomes

The percentage of smokers reached during active implementation was determined by the percentage of smokers who signed up for the intervention out of all smokers admitted to the units during the study period. Data from implementation process measures were juxtaposed with the absolute reach data to identify explanations for variation in reach over time. Using self-reported location data, the reach of participants was also compared between units by phase. The percentages of participants who sent stop messages, reported locations, and replied to a request for feedback were reported. Qualitative analytic procedures for smoker interviews were the same as the nurse interviews. Statistical tests were calculated using STATA 12.1 (StataCorp LLC) and qualitative analyses using NVivo 11.4 (QSR International).

## Results

### Aim 1. Implementation Program Evaluation at the Unit and Nurse Level

#### Characteristics of Units

Four hospital units were identified by organizational nurse leaders (Multimedia Appendix 1, Section A). Organizational readiness scores were similar, with each unit scoring high on the ORCA evidence, context, and facilitation scales.

#### Implementation Fidelity and Adaptability

The length of time for each unit to adopt the posters varied (Multimedia Appendix 1, Section B). In the poster phase, units had an average of 9.6% of posters missing from their walls, with a wide variation between units (range 1.7% to 16.2%). The overall rate of posters missing dropped in the enhanced phase to 1.9%, with a smaller range of missing posters (0.4% to 5.5%) per week. In the sustainability phase, the majority of units (3/4) had a lower rate of missing posters than during the initial poster phase.

Two units hung the posters immediately in week 1, while the other units waited until week 3 and week 5. Stakeholders noted that competing unit priorities and lack of available personnel created barriers to adoption. Stakeholders decided education sessions were to be held during preexisting nurse staff huddles in a common work area on the units. Nurse managers independently facilitated education sessions for their staff nurses: units in one hospital site (units 2 and 4) independently facilitated 7 and 8 additional sessions each, and units in the other site (units 1 and 3) independently facilitated two each.

Unexpected barriers to poster implementation arose, including rooms with contact precautions and wall painting. To overcome these barriers, nurse managers requested business cards containing the poster graphic; these cards were then inserted into standard informational packets given to patients upon admission. In the enhanced phase, manager feedback was integral in creating a visual cue, or cue card, reminding nurses to introduce the intervention and follow the 3-step protocol. Stakeholders identified a need for more nurse engagement beyond training (Multimedia Appendix 2). Tent cards to reiterate training (week 20), paper surveys to counteract low institutional email use (week 25), and feedback boards displaying the unit’s success in introducing the intervention to patients (week 28) were all placed in nurse break rooms. Nurse unit managers did not suggest further adaptations during the sustainability phase.

Totaling just US $2925 overall, this implementation program was very low cost (Multimedia Appendix 3). Even with potential time cost, stakeholders did not identify any additional costs related to the program or implementation beyond materials supplied to the unit. Initial fixed costs of laminated posters and affixation materials were more than three-quarters (US $2240, 76.6%) of the total cost; maintenance costs were negligible. After initial fixed costs were spent, the 60-week program cost US $6.63 (US $683.28/103) per smoker reached and US $8.33 per smoker engaged (US $683.28/82).

#### Nurse Experience With the Program

Nurse survey data, collected at baseline (just after training) and follow-up (at the end of active implementation), showed that nurses overestimated patient and technology barriers to smokers signing up for the intervention compared with nurse report at the end of the enhanced phase (Table 1). One barrier, patient motivation, was severely underestimated (anticipated: n=8, 26%; actual: n=17, 53%).

Program feedback is reported (Multimedia Appendix 4). The number of patients that nurses introduced the program to varied widely, from 1 to 20 people (median 3). Acceptability of the program was indicated, as nurses either strongly agreed or agreed that they would recommend the program to other health care professionals. A perception emerged that the program was a success.

I think that it went very well, that the staff were much more engaged than I thought they were gonna be and that it went really well.

Nurse manager

**Table 1:** Perceived and actual barriers to patient reach by nurses in the enhanced phase.

Barriers to patient reach^a^		Anticipated barriers at baseline (n=31), n (%)	Reported barriers at follow-up (n=32), n (%)	Percentage difference^b^
**Patient characteristics**				
	Language barrier	15 (48)	5 (16)	–32.8
	Patient cognition barrier	13 (42)	3 (9)	–32.5
	Older age of patient	6 (19)	3 (9)	–9.9
	Patient on other substances and a barrier to interest in quitting tobacco	8 (26)	8 (25)	–0.8
**Technology issues**				
	Patient does not have phone	10 (32)	5 (16)	–16.7
	Patient concerned about charges for text messages	8 (26)	3 (9)	–16.4
	Patient does not text	6 (19)	4 (13)	–6.8
	Patient left phone at home	5 (16)	3 (9)	–6.7
**Motivation**				
	Patient not motivated to sign up	8 (26)	17 (53)	27.3

^a^Barriers are organized by largest differences in percentages by category.

^b^Percentage of barriers anticipated at baseline subtracted by the percentage of barriers reported at follow-up.

**Figure 2:**
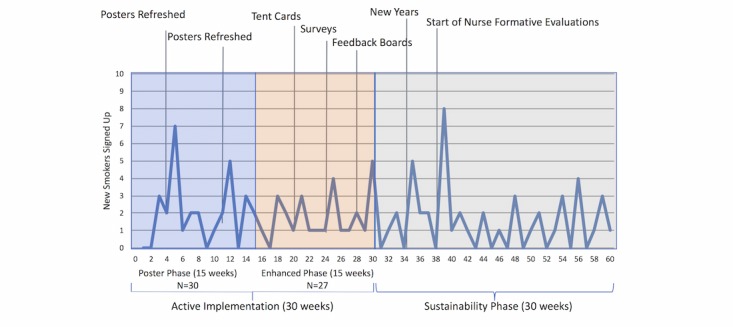
Reach of the Technology Inpatient Program for Smokers intervention by week and phase.

### Aim 2. Impact of the Implementation Program on Smoker Level Outcomes

A total of 103 smokers signed up for the texting intervention over a 60-week period, with varying implementation strategy intensity in all 3 phases (Figure 2). During active implementation, 57 smokers signed up out of a potential 783 smokers admitted to the units (7.3% reach). In the initial poster phase, lasting 15 weeks, 30 smokers signed up. There were large fluctuations by week, and some of the fluctuations mirrored the times when nurse managers took action to facilitate the intervention. For example, during week 4, a unit refreshed their posters, and in week 5, another unit hung their posters for the first time, with a subsequent spike in reach of smokers following these events. In all, 7.1% (30/421) of patients identified as smokers in the EHR signed up for the program (30/328, 9.1% of everyday smokers).

During the nurse staff–facilitated 15-week enhanced phase, 27 smokers signed up. Several peaks in reach during weeks 21, 25, and 30 followed new implementation strategies. Consistent sign-up, with at least one smoker in 14 of the 15 weeks, improved upon the poster phase (11/15 weeks). A total of 7.5% (27/362) of patients identified as smokers in the EHR signed up for the program (27/285, 9.5% of everyday smokers). Throughout the 30-week sustainability phase, 46 smokers signed up, with sign-up steadily decreasing over time (weeks 31-45: mean 1.7, weeks 46-60: mean 1.3). Reach increased in weeks following the start of the New Year (week 34) and following staff interviews (week 38).

**Figure 3:**
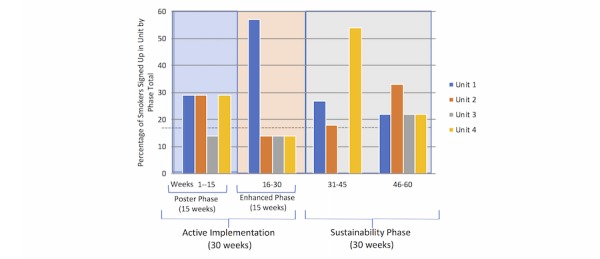
Reach of the Technology Inpatient Program for Smokers intervention by unit and phase. Analysis included only participants who reported location (N=41). Missing responses due to no response (N=57), smokers not on a hospital unit (N=3), and smokers who were unsure what unit they were on (N=2) are assumed to be at random. Mean new smokers signed up for all units over all phases is 2.6 smokers, represented by a dashed line.

Reach of the technology by site was also examined. Analysis was performed with smokers who reported their location (46/103, 44.7%) and excluded several participants not on a hospital unit (3/103) or who were unsure what unit they were on (2/103), for a total of 39.8% (41/103) responses used (Figure 3). In the enhanced phase, one site (unit 1) accounted for over half (8/14, 57.1%) of smokers enrolled, whereas an alternate unit (unit 4) enrolled over half of smokers (6/11, 54.5%) in the first half of the sustainability phase as well (weeks 31-45).

Overall, 79.6% (82/103) of participants completed the full 2-week duration of the intervention (Table 2). Engagement increased from the poster phase (22/30, 73.3%) to the enhanced phase (23/27, 85.2%) when nurse facilitation was initiated. A similar pattern occurred for smoker response to two-way text questions. Responsiveness to patient location more than doubled from poster phase to enhanced phase (2.2, 59.3/26.7) and patient responsiveness to an inquiry for feedback almost quadrupled (3.9, 65.2/16.7). For all measures, engagement declined steadily in the sustainability phase but did not drop to initial poster phase levels.

In follow-up interviews with smokers, text messages were described as easy to read and understandable to all smokers (Multimedia Appendix 5). The majority of interviewed smokers reported that the program made them more serious about quitting. Two-thirds of smokers interviewed reduced their cigarette consumption considerably while using the mHealth intervention, with 2 smokers quitting entirely during and following intervention use. Two smokers were signed up by a family member, a patient was signed up by her sister, and a visitor was signed up by her significant other. The latter two both reported that their family member took the smoker’s phone and texted the number on the poster. Acceptability of the text messages was indicated as smokers strongly agreed or agreed that they would recommend the text messages to others.

**Table 2:** Technology Inpatient Program for Smokers mHealth intervention engagement (main outcome) by smokers.

Phase and total reach	Weeks	Smoker response to two-way text questions during 2-week mHealth intervention		Main outcome (smoker engagement in the mHealth intervention)	
		Responded with location, n (%)	Responded to feedback inquiry, n (%)	Completed full 2-week mHealth intervention, n (%)	Disengaged (did not complete full 2-week mHealth intervention), n (%)
Poster (n=30)	1-15	8 (27)	4 (17)	22 (73)	8 (27)
Enhanced (n=27)	16-30	16 (59)	15 (65)	23 (85)	4 (15)
Sustainability (n=26)	31-45	13 (50)	14 (58)	22 (85)	5 (15)
Sustainability (n=20)	46-60	9 (45)	6 (35)	15 (75)	5 (25)
Total (N=103)	N/A^a^	46 (45)	39 (44)	82 (80)	21 (20)

^a^Not applicable.

## Discussion

### Principal Findings

We successfully completed a two-site implementation of the TIPS program. This program was readily sustained over 60 weeks. To our knowledge, our mHealth intervention is the first Quit Smoking texting system to be offered to patients in hospital units for continued use after discharge. Our findings support the feasibility of engaging smokers to adopt a texting system during the forced abstinence of their inpatient stay. As a light-touch, low-maintenance implementation program, TIPS resulted in an average of 1.7 smokers engaged in the texting system every week. Unexpectedly, nurse-facilitated delivery of the intervention during the enhanced phase did not lead to an increase in the number of smokers adopting the text message intervention. However, as hypothesized, of the smokers who did adopt during this phase, the majority sustained 2-week engagement and at a higher rate than those who adopted during the poster phase.

Adoption of TIPS was sustained over time. While implementation fatigue is often a barrier to consistent evidence-based practice implementation in hospital settings [36], TIPS was well sustained. Organizational theory supports middle managers as key to staff engagement [37] and quality improvement integration within the hospital setting [38]. Managers were able to make changes on their floors through simple educational and maintenance processes. This approach was evidently well received by managers, who participated enthusiastically in new strategy creation, took ownership of hanging posters, and undertook externally implemented staff education. While managers reported the program engaged nurses more than other quality improvement initiatives, excitement about the program faded when new adaptations to strategies were not being implemented.

I think they were pretty good about buying in, but it was kind of there was excitement, and then they lost it. There’d be an excitement again, and then they’d lose it, which if we had that magic wand to keep the excitement going, it’d be great.

Nurse manager

These findings suggest that continual reevaluation of implementation efforts and tailoring those efforts to the unique cultures and practices of different units is a key to sustainability.

### Comparison With Prior Results

Over 60 weeks, a total of 103 smokers adopted the mHealth intervention. Implementation fidelity of posters in the patient rooms was a predictor of reach over time. While posters combined with health care staff training have been used in the implementation of evidence-based practices in hospitals in previous studies [39-43], the relationship between fidelity and reach has been largely unexamined. Our findings were consistent with an mHealth tobacco cessation intervention (iQuit) implemented outside of the hospital setting using an intensive recruitment strategy [44]. Still, a minority of smokers admitted to these units during active implementation, identified using the EHR, adopted the text messaging program. Accuracy of the records, exposure of patients who smoked to posters, and patients’ physical or cognitive ability to sign up while hospitalized are unknown using this low-intensity method. Nevertheless, a minority of the smokers who likely could have adopted did. Nurses reported twice the rate of nonmotivated smokers than anticipated.

I think the challenge is getting people to actually want to sign up. But the actual sign-up process is pretty simple.

Nurse

While the hospital is a teachable moment for some smokers [45], others may need additional help getting motivated. Additional content for these motivational phase smokers should be created and tested.

Engagement with the behavioral intervention is a critical component in the efficacy of an intervention, ensuring smokers receive the full benefit of the 2-week intervention [46]. Overall engagement with text messages in TIPS was similar to prior studies, such as iQuit, where 81% of smokers completed an mHealth intervention [44]. TIPS improved upon longer duration studies in which 31.8% [47] and 45% [48] stopped messages early. Smokers may not have had a fully framed understanding of what the messaging entailed with poster-only facilitation, while nurse delivery allowed for clarification and use of behavior change techniques like persuasive argument, which may have led to stronger engagement with the messages [49]. In her behavior change technique taxonomy, Susan Mitchie and colleagues [49] identified core techniques across theoretical frameworks for behavior change, which include persuasive argument, health consequences, and action planning. Physicians and nurses have used these techniques in tobacco cessation interventions in the past, showing an increased likelihood for cessation [45,50]. We highlighted these techniques during short unit-level nurse training sessions for staff.

Prior work has shown that hospitalization is a teachable moment for families as well as patients, providing enhanced motivation to quit or to stimulate quitting attempts [51]. We similarly found the hospital setting to be a teachable moment for visitors of patients. A surprising finding during formative evaluation of smoker experiences was talking to family members of patients who assisted them in signing up or signed up themselves. Thus far, tobacco-using parents of newborns and hospitalized children are the only populations of family members who have been reached with tobacco cessation interventions in the hospital setting [52-54]. Engaging family members of hospitalized adults in tobacco cessation and considering the role of family or visitors as an avenue of reaching hospitalized smokers are gaps in current tobacco cessation interventions which our implementation strategy might be poised to surmount.

### Limitations

There are limitations to our study. A single health system is not fully generalizable to other inpatient settings, although we did see success in units of diverse specialties, structures, and characteristics across two hospital sites. Six months of active implementation is short for staff practice change, yet nurse managers saw exceptionally high staff engagement during that time period. We collected limited smoker data in an effort to avoid burdening smokers and impeding reach. Anonymity may have been a perceived benefit of mobile messages that helped drive participation. For smokers who disengaged early from the intervention, indicating they would like to stop receiving messages, we did not reach out to determine why. While numerous nurses did introduce the program to smokers, we did not ascertain how many smokers were successfully reached and engaged through posters alone versus posters plus nurse facilitation in the enhanced phase [55]. Process measures for nurse implementation, beyond a final count of smokers reached, may need to be developed further determine the pathway between strategy and smoker engagement.

### Conclusions

TIPS is a low-intensity, sustained program engaging inpatient smokers. While our intervention reached a minority of admitted smokers, the results were comparable to intensive and costly intervention strategies in the outpatient setting [44]. As overall rates of smoking decline, smokers become increasingly challenging to reach and engage. Nurses reported that half of the smokers they approached were not motivated to quit, highlighting the necessity to infuse materials and nurse training with motivational phase-specific strategies. Interestingly, patient family members helped smokers adopt the intervention and even signed up themselves, suggesting an intriguing new public health strategy for using the hospital setting to teach visitors as well as patients. Smokers cluster in social networks, so interventions that can reach and engage both the patient and family may be an exciting innovation. Continued testing of strategies to sustain nurse engagement in facilitation of evidence-based interventions is needed. TIPS represents an innovative, low-cost, easily disseminated strategy for engaging nurses and reaching patients with behavior change interventions.

## Appendix

Multimedia Appendix 1 Technology Inpatient Program for Smokers adoption and implementation by unit and phase.

Multimedia Appendix 2 Materials to engage nurses in program facilitation.

Multimedia Appendix 3 Program cost evaluation.

Multimedia Appendix 4 Themes from qualitative analysis of program feedback interviews with nurses.

Multimedia Appendix 5 Themes from qualitative analysis of program feedback interviews with smokers.

## Abbreviations

EHR: electronic health record
mHealth: mobile health
ORCA: Organizational Readiness for Change Assessment
PRISM: Practical and Robust Implementation Science Model
RE-AIM: reach, effectiveness, adoption, implementation, maintenance
TIPS: Technology Inpatient Program for Smokers
