# Supplementary material for: Nurse-Driven mHealth Implementation Using the Technology Inpatient Program for Smokers (TIPS): Mixed Methods Study
Source: JMIR Mhealth Uhealth. 2019 Oct 4;7(10):e14331. doi: 10.2196/14331 (PMC6818438; doi:10.2196/14331)
Supplement: Multimedia Appendix 1 [file mhealth_v7i10e14331_app1.pdf]

Multimedia Appendix 1. TIPS adoption and implementation by unit and phase.

**Section A:** Hospital Unit Characteristics							
Phase		Measure	Unit 1[a]	Unit 2[b]	Unit 3[c]	Unit 4[d]	Average
All phases		Number of beds	28	25	40	32	125
Poster Phase		ORCA evidence scale	3.75	4	3.75	3.75	3.81 (SD=.125)
Poster Phase		ORCA context scale	3.91	4	3.68	3.77	3.84 (SD=.141)
Poster Phase		ORCA facilitation scale	3.77	4	3.95	3.77	3.87 (SD=.120)
**Section B:** TIPS Implementation							
Phase	Week	Measure	Unit 1[a]	Unit 2[b]	Unit 3[c]	Unit 4[d]	Overall Average
Poster Fidelity							
Poster Phase	1-15	Percent of posters missing	9.7%	11.4%	16.2%	1.7%	9.6%
Enhanced Phase	16-30	Percent of posters missing	5.5%	0.9%	1.2%	0.4%	1.9%
Sustainability Phase	45	Percent of posters missing	3.7%	7.4%	2.6%	7.4%	5.0%
	60	Percent of posters missing	3.8%	7.4%	2.5%	13.3%	6.5%
Nurse Facilitation							
Poster Phase	1-15	Time to adoption: hanging posters (in weeks)	1	1	3	5	2.5
Enhanced Phase	16-30	Number of sessions by nurse unit manager	2	8	2	7	4.75

[a]Unit 1 is an acute care unit with a high number of cardiac pulmonary patients in Hospital 1.
[b]Unit 2 is a family practice unit with a high number of tuberculosis patients in Hospital 2.
[c]Unit 3 is a surgical transplant unit with a number of gastro-intestinal patients in Hospital 1.
[d]Unit 4 is a medical surgical unit in Hospital 2.
