# Supplementary figures and images for: Nurse-Driven mHealth Implementation Using the Technology Inpatient Program for Smokers (TIPS): Mixed Methods Study
Source: JMIR Mhealth Uhealth. 2019 Oct 4;7(10):e14331. doi: 10.2196/14331 (PMC6818438; doi:10.2196/14331)

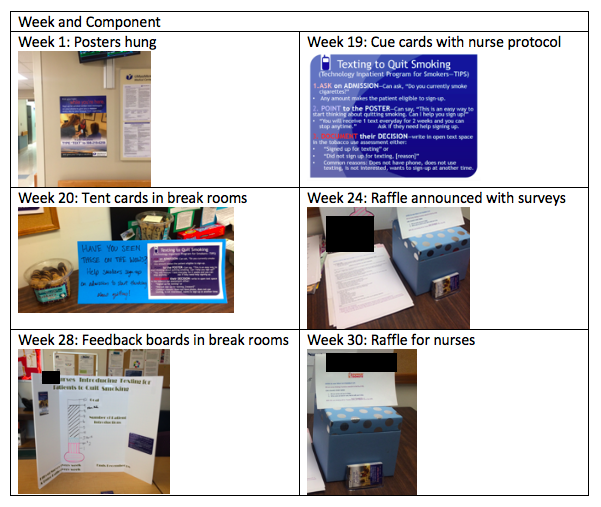

Supplement: Multimedia Appendix 2 [file mhealth_v7i10e14331_app2.png]
