# Supplementary material for: Nurse-Driven mHealth Implementation Using the Technology Inpatient Program for Smokers (TIPS): Mixed Methods Study
Source: JMIR Mhealth Uhealth. 2019 Oct 4;7(10):e14331. doi: 10.2196/14331 (PMC6818438; doi:10.2196/14331)
Supplement: Multimedia Appendix 3 [file mhealth_v7i10e14331_app3.pdf]

Appendix B. Program Cost Evaluation

Material	Cost
**Initial Costs**	
Laminated posters	$2,102.62
Poster tape and strips	$136.27
Total	$2238.89
**Maintenance Costs**	
Poster Phase	
Laminated posters	$137.06
Poster tape and strips	$52.43
Total	$189.49
Enhanced Phase	
Nurse training materials	$40
Laminated cue cards	$210.62
Cue card tape and strips	$18.06
Business card size posters for packet	$66.31
Tent cards and snacks	$100
Feedback boards	$20
Total	$454.99
Sustainability Phase	
Laminated cue cards	$38.80
Total	$38.80
