# Supplementary material for: Nurse-Driven mHealth Implementation Using the Technology Inpatient Program for Smokers (TIPS): Mixed Methods Study
Source: JMIR Mhealth Uhealth. 2019 Oct 4;7(10):e14331. doi: 10.2196/14331 (PMC6818438; doi:10.2196/14331)
Supplement: Multimedia Appendix 4 [file mhealth_v7i10e14331_app4.pdf]

Multimedia Appendix 4. Themes from Qualitative Analysis of Program Feedback Interviews with Nurses.

Feedback from Nurses: Implementation Program	
Likes	*Visually Appealing*<br>*Big*— "I like the poster just because it was big."<br>*Easy to read*— "I like that they were easy to read."<br>*Self-explanatory*— "I like that they're self-explanatory, easy to read."<br>*Visually appealing, looks good*— "I mean, I liked that the posters were visually appealing, and they were available, nurses – for nurses to see them."<br>*An aid to introduction*<br>*Helps nurse introduce texting to patient*— "I think a lotta people use cell phones, so it was nice to be able to say, "Here's this additional alternative." And we had it posted in every patient's room, so I could be like, "It's right up here. This is all the information. You can text this number." So, it was nice to have everything there to show them and to – you're always on your phone anyways, so this is nice to have in front of you."<br>*Can point out information*— "There was some big words on there that were nice to point out, and the number was bigger on there."<br>*Posters remind nurses to ask patients*— "The poster was right in the room, so it reminded me to ask."<br>*Good spot*— "I can't think of any place other than in the – right next to the whiteboard, I think that was a really good spot right there, because patients are always looking up there and a good visual spot."
Dislikes	*Blind to things hanging on walls*— "Sometimes I feel like I just walked by them, so I don't know. I mean, I knew that – I obviously look into my patients' rooms, so maybe I'm just kinda blind to things hanging on the wall now. But I didn't have anybody point them out to me or – so, if I didn't really know to look at it – but the patients are sitting in the room looking at the walls, so I'm sure they can see it."<br>*Unsure if patients got the information*— "We could put something into our welcome packets, something like that. Maybe that might be – make it a little easier for patients to see it more, think about it more."<br>*More education for nurses on the program*— "I don't know if there was one posted in every patient's room, but if not, I think that would be better for the information to be more available to patients. Yeah. I mean, I saw some posters around the nurses' station, and I think some of them were on the WOWs, too, but I didn't attend any education session about it. So, it was kinda like all this information was around me, but I didn't really know how to use it or get that information to patients.
Changes	*Two languages needed, Spanish needed*— "They should have it two language or a different language, 'cause we have different diversities"<br>"No, I liked the whole thing, just the – like I said, the language thing, cause one of my patients was Spanish-speaking. She's like, 'I don't understand, and I don't wanna talk to someone.' So, I said, 'Well, maybe a family member could help you and help translate.' She said, "'OK. Just leave it there.'"<br>*Needs to say more about smoking*— "They might not even read the rest of it unless there's something on top said, 'Stop smoking,' or, 'This will help you to stop smoking.' They might not even pay attention to the poster and read the small print."<br>More information needed with poster— "More towards the patient than us. The poster's great. That's enough for us, but obviously the patients, they like to have their own stuff to read."<br>*Update the poster, once you read it, it will not be re-read*— "The only thing is usually once you read something on a poster or anywhere or – usually we don't reread it again. So, I don't know if there's different things that can be updated for it if it's – if the poster is kept there for a while. I'm not sure if something else could be put on there just to keep our attention to it."
Feedback from Nurses: Text message program	
Likes	*Simple and easy to use*— "I liked the text form. I like that it was simple and easy to use, too."<br>*Promote to keep trying to quit*— "Well, I like the texting part of it. I like that you guys … send a message to kinda promote them to keep trying to stop smoking and all that."

	Reminds smoker to address smoking— "It's like they kinda forgot about it, especially when we ask them at admission. They have so much on their mind … And then when they get the message, they're like, 'Oh, wait, yeah, OK. I remember this.' So, I thought that was a good reminder to them."
Dislikes	*Superficial, "fun fact" level*— "The texts received, the daily texts… seem to be a little light or a little more superficial, as in, "This is a fun fact," and left it at that.<br>*Languages other than English: Spanish language*— "Sometimes we have other patients that speak things other than English. Is there a Spanish-language one…?<br>*Some patients do not want daily text messages*— "Maybe just ask patients throughout if they want to receive a text every day, 'cause not everybody probably want to text every day. So, if that was optional, …it could look more appealing to them, 'cause not everybody wants to get every day text [messages]… [They are] refusing it that way.<br>*Not well understood*— "I did think that was very motivational, I'm not sure if people understood it very well, the patients."
Changes	*Cost to patients*— "I don't know if it cost the patients anything to use it. They had some questions for me that I feel like I couldn't answer."<br>*Kind of messages sent*— "[Patients had] questions like, 'Well, are they gonna text me? What're they gonna send me?' Yeah, 'cause everybody was asking, 'Well, what does it do? What does it tell me?' I don't know. I don't know what kinda messages they send you. I just know that it's something that will encourage you to quit smoking.

TIPS nurse interview data collected after active implementation.
