# Supplementary material for: Nurse-Driven mHealth Implementation Using the Technology Inpatient Program for Smokers (TIPS): Mixed Methods Study
Source: JMIR Mhealth Uhealth. 2019 Oct 4;7(10):e14331. doi: 10.2196/14331 (PMC6818438; doi:10.2196/14331)
Supplement: Multimedia Appendix 5 [file mhealth_v7i10e14331_app5.pdf]

Multimedia Appendix 5. Themes from Qualitative Analysis of Program Feedback Interviews with Smokers.

Feedback from Smokers: Text message program	
Likes	*Convenient and conveniently timed*— "I mean, receiving with text messages is about the easiest way of communicating that you can really come by, so I didn't find any of it a waste of my time"; "It was convenient. They were conveniently timed." "They would sometimes come at a time… when I felt like I want a cigarette. And then I'd get a text message. So, I don't know if it was just good timing or just the universe on my side at that time, but it always came at a good time. I enjoyed getting them."<br>*Informative*— "I liked them, because I feel like they're informative."<br>*Positive*— "I really did think they were extremely helpful, and you guys were really positive about it, and you guys gave really, really good reasons why I shouldn't smoke. It was really good."<br>*Convicting messages*— "So, convicting in a good way, unless you can come up with a better adjective, but it made you stop and think, so I guess that those are good things instead of just continuing on with the behavior."<br>*New platform*— "I think I like the fact that it was a texting program, which was a new platform for me for trying something new and trying to quit something. So, I figured it was kind of a new – using technology, and let's kind of explore and see what it's about."<br>*Constant reminder*— "I liked getting them, 'cause it was like a constant reminder. "
Dislikes	*Information known*— "There was a lot of information that I had already known, and I was hoping for something that would jar me to say, "Oh, my gosh." But they were things that I had heard before. So, I liked getting them, 'cause it was like a constant reminder. I didn't know when they were gonna come. And then some of the information was repeats of what I had previously been informed of."
Changes	*Add encouraging messages*— "I thought a lot of 'em were fact-oriented, which was good, but perhaps encouragement as well, so encouraging text messages or something positive, more positively focused."<br>*Add web links for more information*— "Maybe make them interactive, as if there's links that you can click on for more information."<br>*Send earlier in day*— "I just kinda wish they were a little bit – instead of late in the day, I wish they were early in the morning, only because it would give me more motivation to start off my day reading a text message on the disadvantages of smoking versus it being at the end of the day."<br>*Send more messages*— "I wish I'd gotten more throughout the day, but it was very helpful."<br>*Increase message frequency feature*— "I definitely liked the attitude and the wording. If I could change anything about it, I would be like – maybe send them to them if they sent you, 'I'm stressed out. I need something to get me through this. I need it right now, but send me a message to get me through that.' That's something I'd change, instead of once a day, once they need it, if it was more than once a day.

Feedback from Smokers: Posters	
Likes	*Clear Message* **— "**I just recall the number and it being a clear message, and I said, 'I'll try it.'"<br>"I don't really remember much about the poster. I just remember seeing it, and I'm like, 'OK, that sounds good. Let me try it.'"<br>*Visible*— "had clear fonts that was easy to read, text number to whatever, and you'll start receiving your notifications, which happened immediately"<br>"It was definitely visible. I could see it from across the room. "<br>"Yes, because they were right next to – this particular poster was right underneath the TV, so there was no escaping it. Every time I looked at the TV, the poster was there."
Dislikes	*Poster too small*— "To be honest, they were very – they're on a normal 8.5-by-11 paper, and I missed it. So, maybe having something a little larger would be helpful."<br>*Didn't take it seriously*— "I didn't take them seriously personally, 'cause I didn't think it was a real thing. I thought it was maybe one of those ad spam things, but you guys were really helpful."

Changes	*More serious health message*— "Maybe how seriously bad it affects your life and your health, 'cause they have those commercials about the people with the holes in their throats. Those things are scary. Those definitely would scare me, but you guys weren't like that. You were more friendly about it, but something in the middle of that, not something that would scare you to death but, I don't know, something more in between."<br>*Advertise more throughout hospital*— "I think there needs to be more throughout … the hospital, because walking through the hospital, it's really busy, so maybe making them more visible in more places…I figure it's a great program, and it should be advertised a lot more."

TIPS smoker interview data collected after active implementation.
